# Fronto-Temporal Connectivity Predicts ECT Outcome in Major Depression

**DOI:** 10.3389/fpsyt.2018.00092

**Published:** 2018-03-21

**Authors:** Amber M. Leaver, Benjamin Wade, Megha Vasavada, Gerhard Hellemann, Shantanu H. Joshi, Randall Espinoza, Katherine L. Narr

**Affiliations:** ^1^Ahmanson-Lovelace Brain Mapping Center, Department of Neurology, University of California Los Angeles, Los Angeles, CA, United States; ^2^Department of Psychiatry and Biobehavioral Sciences, University of California Los Angeles, Los Angeles, CA, United States

**Keywords:** major depressive disorder, electroconvulsive therapy, pattern classification, connectomics, functional magnetic resonance imaging

## Abstract

**Background:**

Electroconvulsive therapy (ECT) is arguably the most effective available treatment for severe depression. Recent studies have used MRI data to predict clinical outcome to ECT and other antidepressant therapies. One challenge facing such studies is selecting from among the many available metrics, which characterize complementary and sometimes non-overlapping aspects of brain function and connectomics. Here, we assessed the ability of aggregated, functional MRI metrics of basal brain activity and connectivity to predict antidepressant response to ECT using machine learning.

**Methods:**

A radial support vector machine was trained using arterial spin labeling (ASL) and blood-oxygen-level-dependent (BOLD) functional magnetic resonance imaging (fMRI) metrics from n = 46 (26 female, mean age 42) depressed patients prior to ECT (majority right-unilateral stimulation). Image preprocessing was applied using standard procedures, and metrics included cerebral blood flow in ASL, and regional homogeneity, fractional amplitude of low-frequency modulations, and graph theory metrics (strength, local efficiency, and clustering) in BOLD data. A 5-repeated 5-fold cross-validation procedure with nested feature-selection validated model performance. Linear regressions were applied post hoc to aid interpretation of discriminative features.

**Results:**

The range of balanced accuracy in models performing statistically above chance was 58–68%. Here, prediction of non-responders was slightly higher than for responders (maximum performance 74 and 64%, respectively). Several features were consistently selected across cross-validation folds, mostly within frontal and temporal regions. Among these were connectivity strength among: a fronto-parietal network [including left dorsolateral prefrontal cortex (DLPFC)], motor and temporal networks (near ECT electrodes), and/or subgenual anterior cingulate cortex (sgACC).

**Conclusion:**

Our data indicate that pattern classification of multimodal fMRI metrics can successfully predict ECT outcome, particularly for individuals who will not respond to treatment. Notably, connectivity with networks highly relevant to ECT and depression were consistently selected as important predictive features. These included the left DLPFC and the sgACC, which are both targets of other neurostimulation therapies for depression, as well as connectivity between motor and right temporal cortices near electrode sites. Future studies that probe additional functional and structural MRI metrics and other patient characteristics may further improve the predictive power of these and similar models.

## Introduction

Electroconvulsive therapy (ECT) remains the “gold standard” treatment for severe, treatment-resistant depression, with response rates (50–80%), and response times (<1 month) superior to other currently available treatments (1–3). However, although ECT is generally well-tolerated, the potential for acute (e.g., body aches, confusion) and more protracted (e.g., memory complaints) side effects may impact the decision to initiate or complete ECT treatment. Therefore, identifying prospective biomarkers of antidepressant response to ECT may help patients and their clinicians weigh the potential costs and benefits of ECT, while also contributing to our understanding of the neurobiological mechanisms of this treatment.

During ECT, carefully titrated electrical stimulation is delivered to induce generalized seizures in patients, approximately three times a week for about a month. One might suspect that the lasting effects of repeated generalized seizures on the brain would be widespread and even nonspecific; however, a growing literature suggests that ECT induces plasticity in specific brain regions, including the hippocampus, basal ganglia, anterior cingulate, and prefrontal cortex (4–10). Prospective prediction of ECT response is less studied, but subgenual anterior cingulate cortex (sgACC) gray matter (GM) volume (11) and medial frontal cortex functional connectivity (12) have been recently implicated using machine learning methods. These studies have made important contributions, though several challenges exist in this line of research, including small sample sizes and optimizing selection of statistical models and candidate features (e.g., MRI, demographic, ECT parameters, etc.)

Selecting from among the many available MRI metrics is particularly problematic, because many different kinds of metrics can be derived from the same kind of data that characterize complementary and sometimes non-overlapping aspects of brain function, structure, and/or connectivity. Functional connectomics, for example, can be measured with functional magnetic resonance imaging (fMRI) between two regions, among regions comprising functional networks, or within a single region to characterize local connectivity. Regions and networks can be defined *a priori* based on anatomical location or functional localizers, or can be defined in a data-driven approaches like independent component analysis (ICA) to define resting-state networks (RSNs). Furthermore, calculation of connectivity metrics can be based on similarity of the fMRI timecourse, more complex aspects of network dynamics measured with graph theory, or in the frequency content of the signal (power spectra). Many studies focus on a single approach, though it is likely that a combination of these metrics may relate to clinical response to ECT, and it is unclear whether one or all of them would be most effective in creating prospective predictions of response.

**Figure 1:**
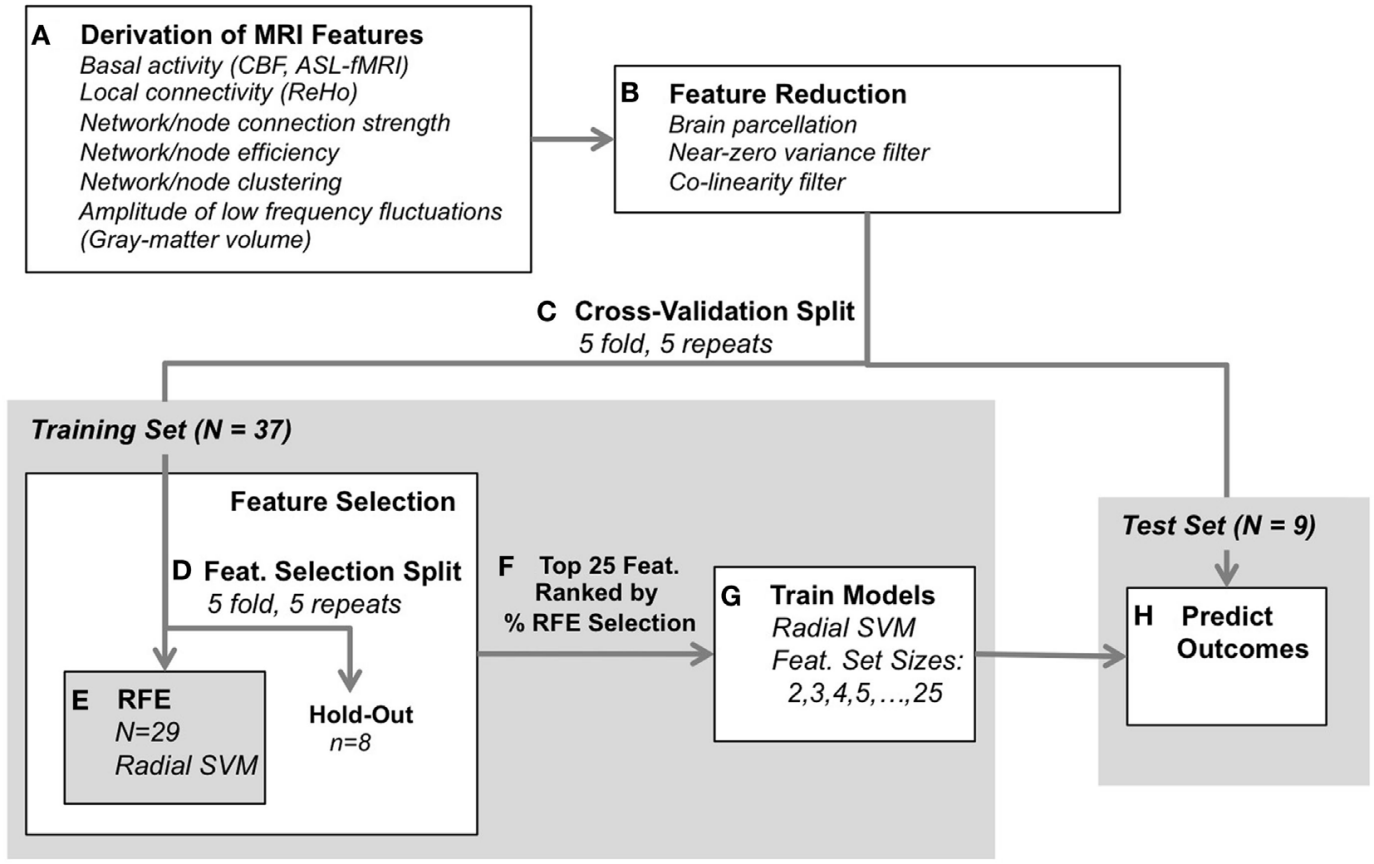
Classification analysis design. **(A)** Functional and structural data were first preprocessed using standard protocols, and metrics were derived to reflect various aspects of brain-network connectivity [blood-oxygenation-level-dependent-functional magnetic resonance imaging (fMRI)], basal activity levels [arterial spin labeled (ASL)-fMRI], and gray-matter volume (structural MRI). **(B)** Next, feature reduction steps derived the average metric for each region of interest (defined by the Craddock atlas), and removed features with near-zero variance and high collinearity. **(C)** Remaining data were split into training and test Sets. **(D–F)** The training set was submitted to feature selection using recursive feature elimination (RFE, radial SVM) to select the 25 most consistently selected multimodal MRI features, validated with 5-repeated 5-fold splits of the training set (resulting in 25 iterations through RFE per each outer cross-validation split). **(G)** A radial SVM classifier was then trained using the training set and the top 25 features ranked by selection consistency. These models included several feature-set sizes, including the top 2 features, top 3, top 4, through top 25 features. **(H)** Finally, each model’s ability to predict electroconvulsive therapy outcome was assessed using the test set. The process was validated across 5-repeated 5-fold cross-validation splits [i.e., **(C)**] for 25 total model predictions for each feature-set size (2–25 features).

In the current study, we assessed the ability of aggregated, multimodal functional MRI metrics to predict antidepressant response to ECT using a data-driven approach. We applied a variety of functional and connectomic analyses to blood-oxygenation-level-dependent (BOLD) and arterial spin labeled (ASL) fMRI data acquired prior to ECT, to create a large set of features that reflected multiple aspects of brain function and connectivity. Structural MRI was also used to derive GM volume for comparison. Depressive symptoms were measured before and after a naturalistic course of ECT, and pattern classification using a radial basis function kernel support vector machine (radial SVM) was applied to predict antidepressant response to ECT. Model predictions and feature selection were validated within a nested 5-fold 5-repeated cross-validation paradigm to ensure consistent performance for our modest sample size (*n* = 46). We hypothesized that a specific subset of these features would be optimally predictive of antidepressant response; therefore, we applied a series of feature reduction and selection techniques in an attempt to identify the most informative functional and connectomic biomarkers of antidepressant response to ECT (Figure 1).

## Materials and Methods

### Participants

Patients (*n* = 46) were assessed twice: before ECT and after index ECT (~4 weeks later). All patients were defined as treatment resistant (failing 2+ prior medication trials) and currently experiencing a DSM-IV-TR-defined major depressive episode. Symptoms of depression were assessed using the Hamilton Depression Rating Scale (17 item) (13), Montgomery Åsberg Depression Rating Scale (14), and Quick Inventory of Depressive Symptoms (15). All procedures were approved by the UCLA Institutional Review Board.

### Electroconvulsive Therapy

Patients volunteered for this research study before initiating a clinically prescribed course of ECT at the UCLA Resnick Neuropsychiatric Hospital. Right-unilateral (RUL) ECT was administered using standard protocols (16) after patients were tapered off all psychotropic medications for a minimum of 48–72 h prior to and for the duration of the ~4-week index series. Some patients who did not respond initially to RUL ECT were transitioned to bitemporal ECT if indicated clinically.

### Image Acquisition

Structural and functional MRI data were acquired using a 3T Siemens Allegra scanner. Continuous ASL images were acquired: 60 volumes (30 label, 30 control), 4 mm × 4 mm × 7.5 mm resolution, 18 axial slices, repetition time 4,000 ms, echo time 16 ms, label time 2,100 ms, post-label delay 1,000 ms, and 95% duty cycle. Blood-oxygenation-level-dependent (BOLD) fMRI images were acquired: 180 volumes, 3.4 mm × 3.4 mm × 5 mm resolution, 34 axial slices, repetition time 2.0 s, echo time 30 ms, flip angle = 70°. During both ASL- and BOLD-fMRI sequences, subjects were resting with eyes closed. A T1-weighted anatomical scan (MPRAGE) was also collected with real-time motion correction (17): (echo times/repetition time = 1.74, 3.6, 5.46, 7.32/2,530 ms, inversion time = 1,260 ms, flip angle = 7°, field of view = 256 mm × 256 mm, 192 sagittal slices, voxel resolution = 1.3 mm × 1.0 mm × 1.0 mm).

### Image Preprocessing: Overview

Arterial spin labeled-fMRI, BOLD-fMRI, and structural-MRI data were preprocessed using standard procedures and target metrics were derived using standard procedures, as described further below. All data were co-registered to MNI space. Voxelwise metrics were averaged within GM regions defined using the Craddock atlas [210 clusters; 202 regions of interest (ROIs) after applying GM mask] (18), separately for each subject. The Craddock atlas is based on a meta-analysis of several functional MRI studies, and offers an independently defined set of ROIs for analysis. Derivation of voxelwise metrics is described below, and included cerebral blood flow (CBF), regional homogeneity (ReHo), fractional amplitude of low-frequency fluctuations (fALFF), GM volume, and BOLD-timecourses used for calculation of graph theory metrics.

### Image Preprocessing: ASL-fMRI

Arterial spin labeled-fMRI images were first corrected for motion (FSL; FMRIB), and then CBF was quantified using the simple subtraction method in ASLtoolbox (19) and averaged across all volumes to yield a single mean CBF image. Mean CBF images were registered to T1-weighted anatomical scans and MNI templates including interpolation to 2 mm × 2 mm × 2 mm voxel size using SPM9 (*Wellcome Trust Centre for Neuroimaging*) and then smoothed with a 6 mm FWHM Gaussian kernel using FSL. Mean voxelwise CBF was averaged within ROIs in the Craddock atlas to yield 202 features per subject.

### Image Preprocessing: BOLD-fMRI

Blood-oxygenation-level-dependent-fMRI images were first preprocessed in FSL, including slice-time correction, motion correction, and high-pass filter (0.01 Hz). Two leading volumes were discarded prior to preprocessing. Spin-history artifacts resulting from interleaved slice acquisition [often correlated with head motion (20)] were removed from voxel timecourses using ICA-based denoising. In brief, ICA was performed for each subject’s BOLD-fMRI data, noise components were labeled manually based on their spatio-temporal profiles, and noise-component timeseries were regressed from the BOLD timecourse using FSL’s regfilt command (20, 21). Finally, preprocessed and denoised images were aligned to each subject’s MPRAGE using FSL, and normalized to MNI standard space including interpolation to 2 mm × 2 mm × 2 mm voxel size using SPM9 and then smoothed with a 6 mm FWHM Gaussian kernel (FSL).

Several functional-connectivity metrics were derived using MNI-normalized BOLD-fMRI data. The REST Toolkit (22) was used to calculate ReHo and fALFF for each voxel, which reflect local connectivity and neurobiologically relevant spectral content of the BOLD timecourse (0.01–0.1 Hz), respectively. Voxelwise ReHo and fALFF metrics were averaged within ROIs of the Craddock Atlas to yield 202 features for each subject (for both ReHo and fALFF). Group ICA was also applied using standard procedures using FSL to derive RSNs in each subject using dual regression. Timecourses of these RSNs were used in calculations of connectivity strength as described below. Graph theory metrics (strength, clustering, and local efficiency) were calculated using the Brain Connectivity Toolbox (23), using both ROIs and ICA-defined RSNs as nodes. “In brief”, “connectivity strength” reflects the correlation between the BOLD-fMRI time courses of two nodes, and was calculated between pairs of ROIs (ROI-to-ROI connectivity strength), between each ROI and RSN (ROI-to-RSN), and between pairs of RSNs (RSN-to-RSN). “Local efficiency” reflects network integration, and was calculated for each ROI as the average shortest path length (i.e., # of contiguously connected nodes) between a given ROI and the thresholded whole-brain network. “Clustering” reflects the degree of separation within a network and was calculated for each ROI. A detailed description of the mathematical derivations and possible interpretations of these and other graph theory metrics can be found in existing methodological literature (23, 24).

### Image Preprocessing: Structural MRI

Structural images were preprocessed in SPM9, as part of the standard tissue segmentation procedure use for MNI-normalization in this package. In brief, images were first corrected for intensity inhomogeneities, and segmented by tissue type (GM, white matter, and CSF) using SPM templates. GM images were then warped to MNI templates, corrected for the amount of deformation applied during normalizing (i.e., Jacobian scaling), and smoothed with a 6 mm FWHM Gaussain kernel (FSL). Final images were thresholded at 0.20 probability of tissue classification as per standard protocols. The resulting voxelwise GM volume metrics were averaged within each of the Craddock Atlas ROIs to yield 202 features per subject.

### Initial Data Reduction and Filtering

After preprocessing and calculation of functional and structural-MRI metrics as described above, our dataset was comprised of 25,311 total features per subject: CBF (202), ReHo (202), fALFF (202), ROI-to-ROI strength (20,301), ROI-to-RSN strength (3,131), RSN-to-RSN strength (465), local efficiency (202), clustering (202), and GM volume (202). In order to simplify interpretation and reduce processing time, we took additional steps to measure and remove redundant features in this dataset, beyond application of the Craddock Atlas as described above (Figure 1A). These data reduction steps and classification analyses (described further below) were applied using standard procedures with the caret Package (25) in R v3.3.2 (www.R-project.org).

First, we removed features with near-zero inter-subject variance. This procedure calculated the frequency ratio for each feature, specifically the frequency of the most prevalent value over the second most frequent value (25). Features with abnormally large ratios were identified as having low variance and removed.

Next, we applied a collinearity filter. In this step, the similarity of features was assessed pairwise, and feature-pairs with *Pearson’s r* > *0.70* were identified as highly collinear. Among these feature-pairs identified as collinear, the feature with least average overall correlation with all other remaining features was retained. These initial feature reduction steps were anticipated to omit redundant and uninformative data not relevant for ECT response (e.g., high collinearity due to neuro-anatomical and/or neuro-functional proximity); therefore, these procedures were not verified using cross-validation.

### Feature Selection and Classification Analyses

After initial data reduction procedures described above, the remaining features were analyzed using the radial SVM algorithm to classify patient participants as ECT responders and non-responders based on patterns in multivariate functional and structural MRI metrics. Using a radial basis function kernel allows fitting of non-linear boundaries (hyperplanes) to separate features, and a strict cross-validation procedure was chosen to avoid over-fitting, which can sometimes be an issue with radial SVM classification. Model performance was robustly validated using a nested 5-repeated 5-fold cross-validation framework, including feature selection using recursive feature elimination (RFE). This process is described in detail below and depicted in Figure 1.

Data reduction steps (described above) yielded 1,844 features each for 46 total patient volunteers. First, patients were evenly divided into “responders” or “non-responders” to ECT, based on the average percent improvement in HAMD-17, MADRS, and QIDS depression scores (even split point was 42.2% reduction in symptoms). This ensured an equally balanced sample for subsequent pattern classification analyses (i.e., *n* = 23 responders and *n* = 23 non-responders), giving a chance classification performance level of 50%.

Next, at the first 5-fold split (cross-validation split, Figure 1C), data were randomly divided into a training set (*n* ≈ 37) and test set (*n* ≈ 9). The training set was submitted to feature selection, to determine the optimal set of predictive features with RFE (26). In RFE, features were ranked according to their estimated predictive value using radial SVMs (and internal 5-repeated 5-fold cross-validation), and the feature set with the best classification performance (area under the receiver operating curve) was chosen. This was repeated for each feature-selection split (Figure 1D), for 5 folds and 5 repeats yielding 25 total feature sets.

The features comprising these RFE-selected feature sets were then ranked based on the consistency with which they were chosen across feature-selection splits, where 100% consistency for a given feature would indicate that it was chosen 25 times (Figure 1F). The top 25 features were then passed to the next step, where the SVMs were trained on the entire training set using these features (Figure 1G). Feature-set sizes were varied, such that model feature-sets included either the top 2 features, top 3, top 4, and so on, resulting in 24 total models. The predictive accuracy of these models were tested on the test set (Figure 1H), and balanced accuracy, sensitivity (to predict response), and specificity [to predict non-responders (NR)] were derived. We chose balanced accuracy (i.e., the average of sensitivity and specificity) to avoid inflating our reported model performance (e.g., if prediction of non-responders was more accurate than for responders). Note that in all cases radial SVM parameters were optimized using the area under the receiver operating characteristic curve metric to maximize balanced prediction of both response and non-response to ECT. This entire procedure was repeated for each cross-validation split (Figure 1C) to yield 25 total sets of final models and associated predictions.

This entire procedure (Figures 1C–H, feature selection and model estimation), including cross-validation and feature-selection splits, was applied both to true “veridical” data to address our hypotheses of interest, and again on identical data paired with randomly shuffled responder/non-responder labels to estimate chance performance. Veridical and random-label data were compared with Welch’s *t* tests, assuming unequal variance and estimating one-tailed *p*-value to address the hypothesis that veridical data would outperform randomly sampled class data. These *t*-tests were performed for all predictions across the 25 cross-validation splits, separately for each feature-set size (i.e., 2–25 features) for balanced accuracy, sensitivity, and specificity. *p*-values were corrected for multiple comparisons using the false discovery rate.

### *Post Hoc* Analyses

Features that were selected with high consistency across cross-validation splits were targeted in univariate analyses *post hoc* to assess each of their relationships with ECT outcome. Linear regression analyses tested for correlations between each feature and mean percent change in depression scores (averaged across HAMD, MADRS, and QIDS), with age as an additional nuisance covariate. Note that results were similar with and without age included in these models. Uncorrected *p*-values are reported for these tests.

## Results

### Demographic and Clinical Variables

Depression scores improved significantly after ECT (*p* < 0.00001 for all scales), with 22 of 42 of patients exhibiting at least a 50% reduction in depression scores and 16 of 43 patients meeting criteria for remission on any of our symptom inventories after the ECT index treatment series. These measures and other clinical variables are displayed in Table 1.

**Table 1:** Sample characteristics.

Sample size	46	
Age, mean (SD)	41.74 (6.78)	
Sex, females/males	26/21	
Diagnosis, unipolar/bipolar	38/8	
Age of diagnosis, mean (SD)	25.07 (6.78)	
ECT leads, only RUL/other	29/17	
Responder/non-responder	20/26	
Number of *tx*, mean (SD)	11.59 (6.78)	
	Pre-ECT	Post-ECT index
	
HAM-17, mean (SD)	24.02 (6.78)	13.21 (7.66)*
MADRS, mean (SD)	37.50 (6.78)	18.60 (11.37)*
QIDS-SR, mean (SD)	19.74 (6.78)	11.00 (5.91)*

*Significant change from pre-ECT values (paired t-test).

### Classifier Performance

**Figure 2:**
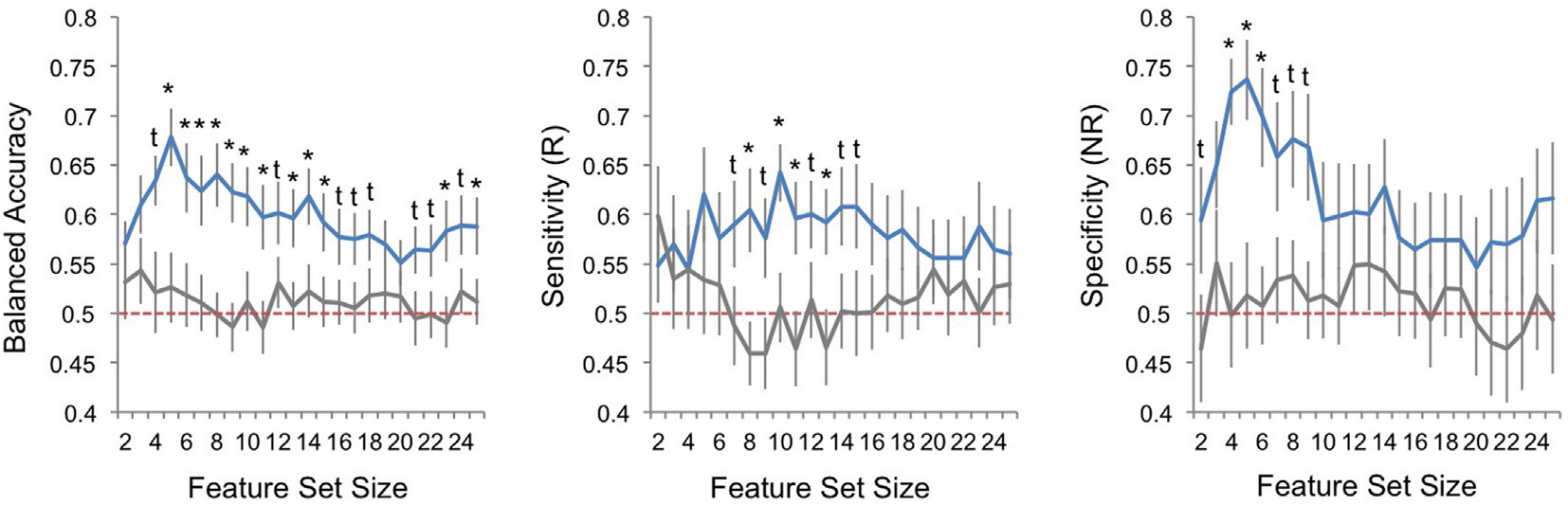
Classification performance across sets of best features. Plots of pattern classification performance are displayed for balanced accuracy (left panel), sensitivity [to predict electroconvulsive therapy (ECT) responders, R; middle panel], and specificity [to predict ECT non-responders, NR; right panel] and all feature-set sizes (i.e., number of ranked features used in each model). Performance is averaged over all 25 outer cross-validation loops; error bars reflect standard error. Blue lines indicate model performances for veridical datasets, and gray lines mark performance for random datasets where responder/non-responder labels were randomly assigned. Red-dashed line marks chance performance (50%). The results of Welch’s *t*-tests comparing veridical and random data are given; asterisks mark one-tailed false discovery rate-corrected *p* < 0.05 and daggers mark one-tailed uncorrected *p* < 0.05.

Performance metrics are displayed for veridical and random data and all models (i.e., feature sets) in Figure 2. Balanced accuracy for veridical data was consistently higher than for identical data with randomly shuffled class labels (responder/non-responder). When considering model performance across feature-set sizes, average balanced accuracy was statistically higher than performance for random-label data, with minimum and maximum averaged performance for veridical data at 58 and 68%, respectively. Sensitivity (i.e., prediction of responders) was slightly lower than specificity (i.e., prediction of non-responders), though both were above chance in a number of model parameterizations (*p*_FDR_ < 0.05). Range of averaged sensitivity across model parameterizations (i.e., across feature-set sizes) was 54–64%; range of average specificity was 55–74%.

### Feature-Selection Results

**Figure 3:**
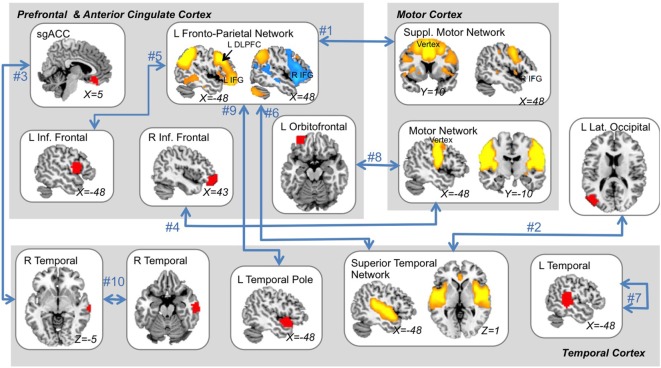
The ten “best” multimodal functional magnetic resonance imaging features. The top 10 features that were most consistently selected across all outer cross-validation splits are displayed. All top features related to functional-connectivity strength among networks (orange and blue regions) and/or single brain areas (red regions) as indicated by blue arrows. A single feature reflected local connectivity strength (i.e., regional homogeneity). Feature rank is displayed next to blue arrows (see also Table 2) and MNI coordinates are given for each slice displayed. L, left; R, right; ACC, anterior cingulate cortex; DLPFC, dorsolateral prefrontal cortex; suppl., supplementary; Inf., inferior.

Features that were selected with high consistency across cross-validation splits were targeted for further analysis. The top 10 most consistently selected features are displayed in Figure 3, and the top 25 are given in Table 2. Two features were selected on every cross-validation split (i.e., with 100% frequency) and included (1) connectivity strength between a left fronto-parietal network and the supplementary motor network and (2) connectivity between a superior temporal network and left lateral occipital cortex. A third feature was selected in all but two cross-validation splits (i.e., with 92% frequency), which was connectivity between sgACC and a right temporal cortex region. Notably, of the top 25 most consistently selected features across cross-validation splits (Table 2), 56% involved temporal regions or networks, 28% involved the left fronto-parietal network and 56% involved prefrontal networks or regions more generally, 32% involved motor or supplementary motor networks or regions near the ECT vertex electrode, and 8% involved sgACC.

**Table 2:** Top-ranked features (most consistently selected across cross-validation splits).

Rank	% Select	Metric	Region/network 1		Region/network 2	
				
			Index	Description	Index	Description
1	100	Connectivity strength	NET1	L Fronto-Parietal Network	NET2	Supplementary Motor Network
2	100	Connectivity strength	NET3	Superior Temporal Network	ROI1	L Lateral Occipital Cortex
3	92	Connectivity strength	ROI2	Subgenual Anterior Cingulate Cortex	ROI3	R Superior Temporal Cortex
4	76	Connectivity strength	NET4	Motor Network	ROI4	R Inferior Frontal Gyrus
5	76	Connectivity strength	NET1	L Fronto-Parietal Network	ROI5	L Inferior Frontal Gyrus
6	64	Connectivity strength	NET1	L Fronto-Parietal Network	NET3	Superior Temporal Network
7	60	Regional homogeneity	ROI6	L Superior Temporal Cortex	n/a	n/a
8	56	Connectivity strength	ROI7	R Orbitofrontal Cortex	NET4	Motor Network
9	56	Connectivity strength	NET1	L Fronto-Parietal Network	ROI8	L Temporal Pole
10	56	Connectivity strength	ROI1	R Superior Temporal Cortex	ROI9	R Inferior Temporal Cortex
11	56	Connectivity strength	NET1	L Fronto-Parietal Network	ROI10	R Superior Parietal Cortex
12	52	Connectivity strength	ROI11	R Rostral Inferior Temporal Cortex	NET4	Motor Network
13	44	Connectivity strength	ROI12	L Dorsolateral Prefrontal Cortex	ROI11	L Superior Parietal Cortex
14	44	Connectivity strength	NET1	L Fronto-Parietal Network	ROI11	L Superior Parietal Cortex
15	44	Connectivity strength	NET4	Motor Network	ROI3	R Superior Temporal Cortex
16	44	Connectivity strength	ROI13	L Suppl. Motor Cortex	ROI14	L Dorsal Premotor Cortex
17	40	Connectivity strength	ROI11	L Lateral Parietal	ROI15	L Lateral Occipital Cortex
18	36	Connectivity strength	NET1	L Fronto-Parietal Network	ROI16	L Superior Temporal Cortex
19	36	Connectivity strength	ROI2	Subgenual Anterior Cingulate Cortex	NET3	Superior Temporal Network
20	32	Connectivity strength	ROI17	Dorsomedial Prefrontal Cortex	ROI18	R Rostral Sup. Frontal Sulcus
21	32	Connectivity strength	ROI5	L Inferior Frontal Gyrus	NET5	Fronto-Temporal Network
22	32	Connectivity strength	ROI15	L Lateral Occipital Cortex	NET6	Anterior Temporal Network
23	28	Regional homogeneity	ROI19	R Inferior Temporal Cortex	n/a	n/a
24	28	Connectivity strength	ROI20	L Rostral Superior Frontal Gyrus	NET7	Medial Occipital Network
25	28	Connectivity strength	NET4	Motor Network	ROI21	L Post. Sup. Temporal Cortex

*R, right; L, left; Sup., superior*.

*Note that “Index” indicates the identity of each network (NET) or region of interest (ROI) comprising the given feature; identical indices indicate identical regions or networks*.

**Figure 4:**
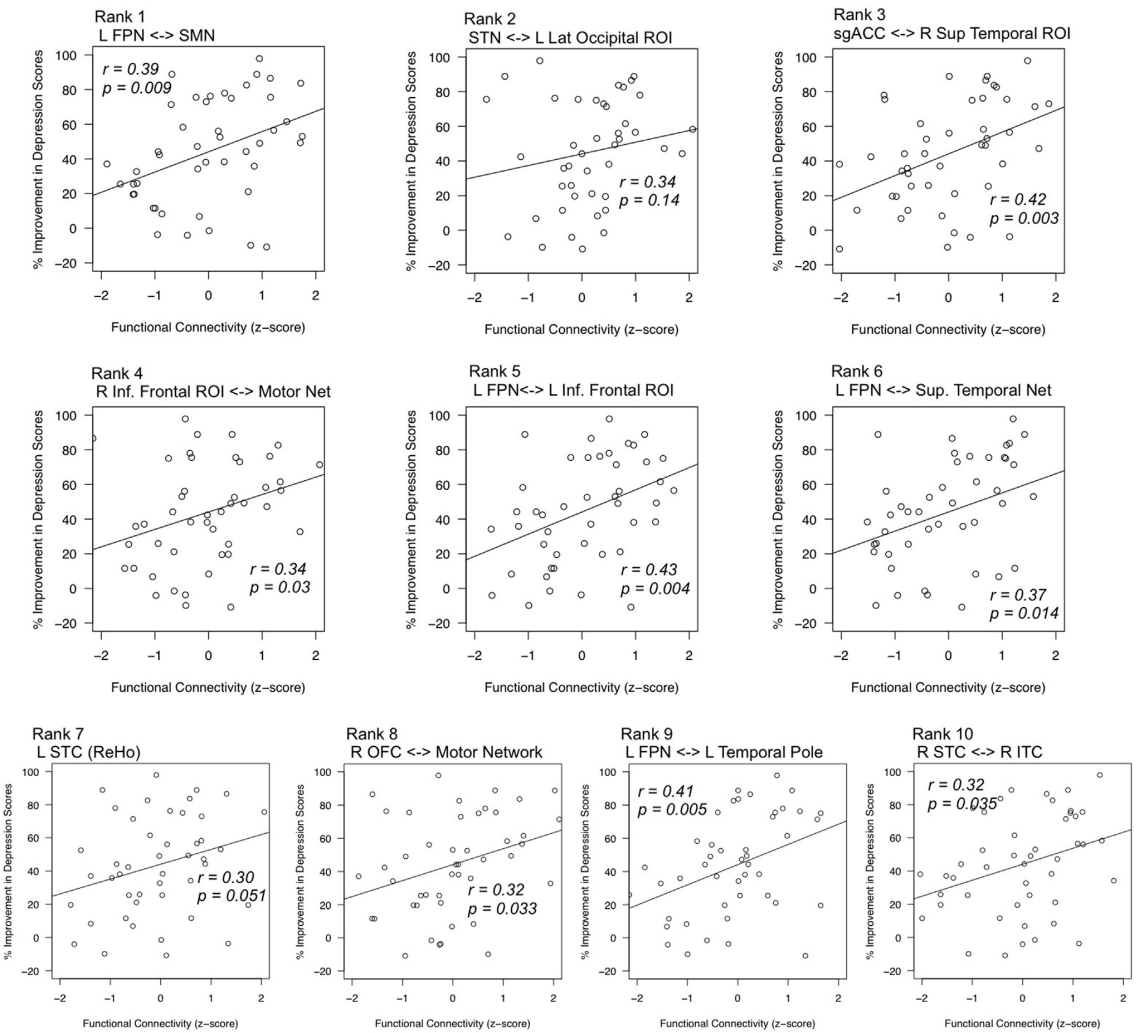
Correlations between top features and mean change in depression scores. Statistical tests *post hoc* confirmed relationships between highly robust features (*x*-axes) and improvement in depression scores (*y*-axes). Feature rank was determined by the consistency of feature selection across cross-validation splits. Pearson’s *r* values are given for each respective regression line, and *p*-values reflect the results of regression analyses with age as a nuisance factor. L, left; R, right; FPN, fronto-parietal network; SPN, supplementary motor network; STN, superior temporal network; sgACC, subgenual anterior cingulate cortex; inf., inferior sup., superior; STC, superior temporal cortex; OFC, orbitofrontal cortex; ITC, inferior temporal cortex; ReHo, regional homogeneity.

*Post hoc* analyses estimated correlations between each of these features and changes in depression scores after ECT. All features exhibited positive correlations between connectivity strength and change in depression scores, such that volunteers who responded to ECT were more likely to have increased connectivity (all but one *p* < 0.05). Corresponding scatter plots are displayed in Figure 4.

**Figure 5:**
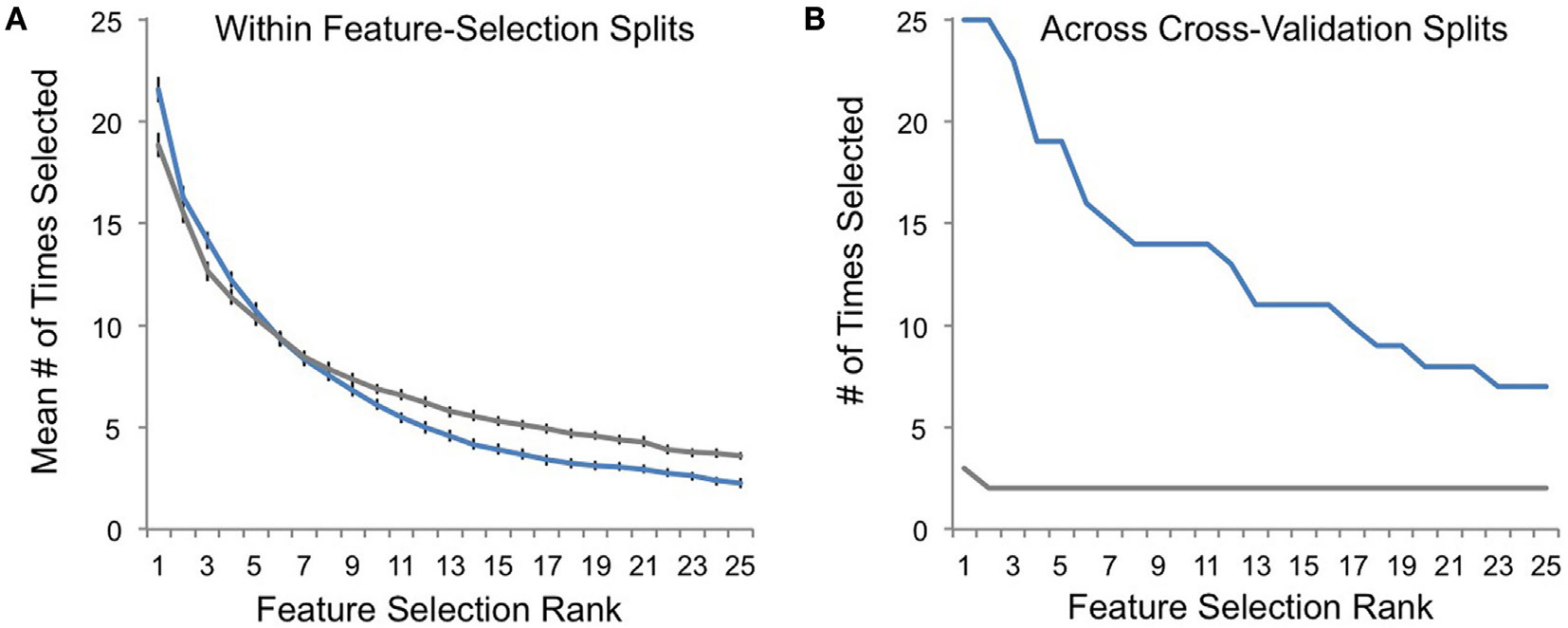
Feature-selection consistency. **(A)** The average consistency within each round of recursive feature elimination (RFE) feature selection is plotted on the *y*-axis for veridical data in blue and for randomly assigned responder/non-responder labels in gray. Here, a value of 25 indicates that the top-selected feature (e.g., rank 1) was selected with 100% consistency within RFE feature selection (Figure 1D) for all cross-validation splits (Figure 1C). **(B)** The frequency with which top-ranked features (Figure 1F) were selected across cross-validation splits is plotted on the *y*-axis for each of the most consistently selected features on the *x*-axis (ranked by number of times selected across cross-validation splits).

Finally, we performed *post hoc* analyses comparing feature-selection consistency for veridical and random-label data. Within each round of feature-selection splits (Figures 1D–F), there was a high degree of feature-selection consistency for both veridical and random-label data sets. In other words, the RFE procedure was consistently choosing feature sets that best predicted class labels within each training set regardless of whether those labels were randomly shuffled (Figure 5A). However, across cross-validation splits, feature-selection consistency was only high for veridical data, and was correspondingly quite low for data with randomly shuffled labels (Figure 5B). This suggests that although the RFE feature-selection procedure characterized each single training set well, the predictive power of the features selected only generalized well to the test set for veridical data, not random data. This speaks to the robustness of our nested-cross-validation design to enhance the potential for generalizability of our models.

## Discussion

In this study, we provide evidence that functional connectivity of specific frontal and temporal regions measured prior to treatment can predict antidepressant outcome of RUL ECT in patients with severe, recurrent depression. These results were robust across 5-repeated 5-fold cross-validation, and against randomly shuffled data as well. Notably, connectivity strength in networks including two regions highly relevant to depression, the left dorsolateral prefrontal cortex (DLPFC) and sgACC, were consistently identified as informative features in our models. The left DLPFC and sgACC are targets of other neurostimulation treatments for depression (TMS and DBS, respectively); our data indicate that pretreatment connectivity of these regions may be important in determining the success of ECT. Taken together, our results indicate that the strength of pretreatment functional connectivity between depression-relevant regions and RUL-electrode-adjacent regions may strongly influence the probability of positive ECT outcome. In the following sections, we discuss our results with respect to existing literature and their potential impact on future research.

### Pretreatment Fronto-Temporal Connectivity Predicts ECT Outcome

In RUL ECT, alternating current is passed between two electrodes, one at the vertex (top) of the head and another at the right temple (27). This alternating current elicits highly coordinated brain activity, which ultimately results in a generalized seizure where seizure activity occurs in all (or most of) the brain. Although the end result is a generalized seizure, presumably the highly coordinated brain activity that results in that seizure occurs, at least initially, in regions along the path of electrical current that travels between the vertex and right-temple electrodes. Indeed, our results indicate that the functional state of brain regions near these electrodes prior to treatment, particularly with respect to their connections with depression-relevant regions like sgACC and left DLPFC, may determine whether ECT is successful in reducing the symptoms of depression.

Networks involving motor and supplementary motor corticies were consistently chosen as predictive features by our models. Both these motor networks were comprised of lateral and medial motor cortical regions located near the vertex RUL electrode. These findings support previous ECT studies, which have implicated an RSN similar to our supplementary motor network [van Waarde et al. (12)] and GM near motor and supplementary motor cortex [Jiang et al. (28)] in prospective prediction of ECT outcome using machine learning. Cortical thickness near motor cortex was also found to predict ECT relapse in a recent report as well [Wade et al., in press (29)]. Notably, however, connectivity between these motor networks and brain regions strongly linked to depression and antidepressant response in previous studies were most influential in our models, for example, the left DLPFC and right inferior frontal gyrus (IFG) (30–32). Indeed, functional connectivity strength measured between the Supplementary Motor Network and the Left Fronto-Parietal Network (including left DLPFC and right IFG) was consistently selected as an important predictive feature in 100% of cross-validation splits. This indicates that RUL ECT may work, at least in part, by influencing depression-relevant prefrontal regions through coordinated/seizure activity in motor regions near the vertex electrode. Alternatively, involvement of supplementary motor regions in particular could reflect links to motor planning (33, 34), which is a core deficit of major depression.

A large number of features consistently selected in our models were also comprised of temporal cortex regions and/or networks, located near the temporal RUL electrode. This corresponds nicely with existing literature pointing to a particular role for medial temporal (e.g., the hippocampus) and other temporal regions in ECT and ECT outcome (5, 7, 10, 35, 36). Structural plasticity of the hippocampus and surrounding medial temporal lobe structures after treatment is a common finding in ECT research (7, 10, 11, 35, 36), and previous reports also indicate that ECT may alter connectivity between temporal cortex and the hippocampus (36). Our data add to these results by indicating that functional connectivity within temporal cortex regions prior to treatment may influence ECT outcome.

Fronto-temporal connectivity was also consistently identified as an important feature in our study. Strength of functional connectivity between temporal cortex and sgACC was selected with a high degree of consistency (92% of cross-validation splits), and connectivity between temporal regions and the Left Fronto-Parietal Network was identified in two separate top-ranked features. Again, this suggests that pretreatment connectivity between electrode-adjacent regions in temporal cortex and depression-relevant regions like sgACC, DLFPC, and other prefrontal regions may influence how patients respond to ECT. This also corresponds well with previous studies linking sgACC with ECT outcome. For example, Redlich et al. (11) recently demonstrated that sgACC GM volume may predict ECT outcome using machine learning (11), and Argyelan et al. (37) reported that functional connectivity (fALFF) in sgACC was correlated with ECT outcome at baseline (37). It may seem counter-intuitive that pretreatment connectivity within frontal regions relatively far from ECT electrodes should influence ECT outcome. However, we propose that, taken together, our results indicate that pretreatment connectivity between these depression-relevant regions and electrode-adjacent regions may indeed influence ECT outcome.

### Interpreting Classification Performance and Potential Clinical Impact

Interpreting the translational value of complex multimodal MRI analyses like these can be challenging, particularly because there are several different metrics that can be used to describe classifier performance. Our highest performing model parameterization had a mean balanced accuracy of 68% (SD: 14.53%; range: 38–100%; feature-set size = 5), sensitivity to predict response of 62% (SD: 23.71%; range 25–100%), and specificity to predict non-response of 74% (SD: 20.34%; range 20–100%). We chose these metrics because we felt it important to focus on models that performed optimally for both responders and non-responders. However, performance can also be interpreted using positive and negative predictive values, which may have more direct clinical value. For example, performance for our “best” model parameterization corresponded with an average positive predictive value of 0.73, meaning that if these models labeled a patient as a “responder” prior to treatment, the probability that patient would respond to ECT improves from 50 to 73%. Similarly, negative predictive value was 0.67, which means that if these models predicted that a patient would *not* respond to ECT, this would decrease the probability of response from 50 to 33% (i.e., one minus negative predictive value). Note that 50% response rate here was derived based on an even split of our data into equal numbers of responders and non-responders; response rates calculated by more traditional means were higher (Table 1). Taken together, these numbers are promising; however, cross-site validation is needed to determine the generalizability of the predictive value of the connectomic features the current analyses have identified. Multi-site studies on a larger scale that also leverage additional MRI and other metrics (e.g., demographics, clinical features, gene expression, etc.) (28, 29), including fused multimodal features (38), are likely to be even more successful in prospectively predicting treatment outcome, and perhaps even using pretreatment measures to optimize ECT parameters.

### Limitations and Additional Methodological Considerations

A major challenge when applying machine learning in MRI research is that the number of features used to define a predictive model is often several orders of magnitude larger than the sample used, which leads to over-fitting of the current dataset and, correspondingly, a decreased likelihood of generalization to new patient cohorts. We addressed this issue in several ways. First, we applied feature reduction and selection procedures to minimize the number of features used in our predictive models. Second, we applied nested cross-validation to attempt to minimize over-fitting our models to our current dataset. Indeed, comparing Figures 5A,B demonstrate the success of some aspects of our approach: although feature-selection consistency was high for both random and veridical data within the RFE feature-selection procedure, this consistency only generalized to the test set when analyzing veridical, non-random data. This speaks to the potential utility of nested cross-validation when using this feature-selection approach with MRI data to minimize over-fitting.

Note too that our data reduction procedure transformed voxelwise metrics to larger ROIs. In part, this was done to mitigate computational burden, which can be significant. However, this procedure also achieved reduction in noise due through averaging feature metrics across voxels within the ROI, and also saved us from a scenario where single voxels (e.g., at tissue boundaries or other potentially uninformative regions) could be chosen by the classifier as influential predictive features [e.g., as in Redlich et al. (11) or van Waarde et al. (12)]. On the other hand, by using a single ROI size, we made an implicit assumption *a priori* that relevant features would be of a certain size, perhaps limiting contributions of smaller regions, thereby reducing specificity. Future models targeting ROIs in a range of sizes may be better able to capture, for example, hippocampal or basal ganglia contributions that were not selected by our models as being predictive of ECT outcome.

### Conclusion

Here, we make a case for the use of aggregated, multimodal fMRI features in predicting antidepressant response to RUL ECT in severe, recurrent depression using pattern classification. The overall performance of our models was above chance performance (i.e., compared to random-label data) for classification of both responders and non-responders, and was robust to cross-validation procedures. In top-performing models, probability of response increases from 50 to 73% if a patient is classified as responder, and probability of response decreases from 50 to 33% if classified as a non-responder by these models (i.e., positive and negative predictive values). Although models with higher accuracy have been reported previously (11, 12), we would argue that a reduction in probability of response of 17% may be useful to patients and physicians when weighing treatment options. The most consistently selected features in our models encompassing fronto-temporal regions near RUL ECT electrodes; connectivity between these regions and depression-relevant regions like sgACC and left DLPFC were also identified as important. Thus, in addition to the potential clinical utility of these predictive models, the top-ranked features identified also contributed to our knowledge of ECT mechanisms. Going forward, multi-site studies with larger and varied cohorts, targeting responsibly preprocessed MRI and other data will be in an even better position to determine the extent to which these kinds of analyses can further elucidate the mechanisms of ECT and perhaps be used to make more accuracy prospective prediction of successful antidepressant response to ECT.

## Ethics Statement

This study was carried out in accordance with the recommendations of the UCLA Institutional Review Board with written informed consent from all subjects. All subjects gave written informed consent in accordance with the Declaration of Helsinki.

## Author Contributions

KN and RE designed the study. AL, MV, and KN collected and preprocessed MRI and clinical data. AL, BW, and GH designed and applied statistical analyses. All authors contributed to writing this manuscript.

## Conflict of Interest Statement

The authors declare that the research was conducted in the absence of any commercial or financial relationships that could be construed as a potential conflict of interest.
